# Mechanisms of soft tissue and protein preservation in *Tyrannosaurus rex*

**DOI:** 10.1038/s41598-019-51680-1

**Published:** 2019-10-30

**Authors:** Elizabeth M. Boatman, Mark B. Goodwin, Hoi-Ying N. Holman, Sirine Fakra, Wenxia Zheng, Ronald Gronsky, Mary H. Schweitzer

**Affiliations:** 1 Department of Engineering, Wake Forest University, Winston Salem, NC 27101 USA; 2 Museum of Paleontology, University of California, Berkeley, CA 94720 USA; 3 Advanced Light Source, Lawrence Berkeley National Laboratory, Berkeley, CA 94720 USA; 4 Department of Biological Sciences, North Carolina State University, Raleigh, NC 27695 USA; 5 Department of Materials Science and Engineering, University of California, Berkeley, CA 94720 USA; 6 Department of Geology, Lund University, Lund, Sweden; 7 North Carolina Museum of Natural Sciences, Raleigh, NC 27601 USA; 8 Museum of the Rockies, Montana State University, Bozeman, MT 59715 USA

**Keywords:** Palaeontology, Palaeontology

## Abstract

The idea that original soft tissue structures and the native structural proteins comprising them can persist across geological time is controversial, in part because rigorous and testable mechanisms that can occur under natural conditions, resulting in such preservation, have not been well defined. Here, we evaluate two non-enzymatic structural protein crosslinking mechanisms, Fenton chemistry and glycation, for their possible contribution to the preservation of blood vessel structures recovered from the cortical bone of a *Tyrannosaurus rex* (USNM 555000 [formerly, MOR 555]). We demonstrate the endogeneity of the fossil vessel tissues, as well as the presence of type I collagen in the outermost vessel layers, using imaging, diffraction, spectroscopy, and immunohistochemistry. Then, we use data derived from synchrotron FTIR studies of the *T. rex* vessels to analyse their crosslink character, with comparison against two non-enzymatic Fenton chemistry- and glycation-treated extant chicken samples. We also provide supporting X-ray microprobe analyses of the chemical state of these fossil tissues to support our conclusion that non-enzymatic crosslinking pathways likely contributed to stabilizing, and thus preserving, these *T. rex* vessels. Finally, we propose that these stabilizing crosslinks could play a crucial role in the preservation of other microvascular tissues in skeletal elements from the Mesozoic.

## Introduction

Hollow, pliable, and transparent vessel-like structures have been recovered from skeletal elements of multiple fossil vertebrates, including non-avian dinosaurs^1,2^. Their vascular affinities have been supported through the application of varied independent methods to identify endogenous component proteins^3,4^, including collagen, which is not produced by microbes^5^, and elastin, which is vertebrate-specific^6^. Mass spectrometry sequencing of isolated vessels recovered from the cortical bone of a non-avian dinosaur further supported the presence of vertebrate-specific vascular proteins in isolated dinosaurian vessels^7^. The hallmark 67-nm-banding pattern typical of type I collagen has been documented in fossil tissues, following liberation by demineralisation^8^, and the presence of type I collagen in the vascular canals of a ~190 Mya sauropod dinosaur rib was suggested by synchrotron Fourier-transform infrared spectroscopy and Raman analyses^9^.

Various mechanisms, including transition metal-catalysed intermolecular crosslinking of structural proteins^2,10^, have been proposed to explain this unexpected preservation, but experimental testing of these proposed mechanisms has not been widely conducted. Therefore, in this work, our goal was to identify and test for the possible contribution of an explicit set of transition metal-catalysed crosslinking mechanisms to the preservation of vessel-like structures recovered from the compact bone of a *Tyrannosaurus rex* (USNM 555000 [formerly, MOR 555]), to lay a possible foundation for additional studies of preservation mechanisms for other soft tissues recovered from Mesozoic or more recent fossils.

The walls of vertebrate blood vessels are comprised of three distinct layers, the tunica intima (innermost, also identified as the tunica interna), tunica media, and tunica externa (outermost)^11^. These layers can be differentiated morphologically and chemically because of their unique molecular composition. Homotypic type I and heterotypic type I/III fibrillar collagen molecules, both of which exhibit 67-nm-banding character and are vertebrate-specific^5,12–15^, constitute the predominant collagen fraction of blood vessels (as much as 90%), primarily localizing to the tunica media and tunica externa to serve as the structural foundation of the vessel^11,12,16^. Elastin, a helical protein also specific to vertebrates^6^, confers resistance to pressure changes in vascular walls^11^ and is localized primarily to the tunica media and the basement membrane, which separates the tunica intima from the tunica media^17^. Thus, we proposed that these proteins could be detectable in some form if the structures investigated in this work were remnant dinosaur vessels, with chemical signatures diagnostic of their current preservation state.

Both elastin and collagen are identifiable by certain hallmark features constrained by their structure and molecular composition. For example, collagen is a repetitive helical protein with every third residue occupied by glycine^12^, which demonstrates unusual hydroxylation patterns on proline and lysine residues^18^. The 67-nm-banding motif of fibrillar collagen results from a characteristic head-to-toe stacking pattern and offset of adjacent molecule stacks that results from chemical composition and is critical to mechanical performance^12–15^. Elastin is also a highly repetitive helical protein capable of self-assembly, and is comprised of high levels of glycine, proline, and valine^19^. The tertiary structure of both fibrillar collagens and elastin arises from intramolecular crosslinks formed between lysine residues on adjacent tropocollagen and tropoelastin molecules, respectively, and in living organisms, these pathways are mediated by similar lysyl oxidase (enzymatic) mechanisms (Fig. S1)^20,21^.

However, intramolecular (and ultimately, intermolecular) crosslinks can also form by non-enzymatic, and hence unregulated, pathways, particularly as tissues age^12,22,23^. Such pathways have also been studied in association with atherosclerotic plaque formation, changes in hormones, and glucose regulation, among others^22–24^. The presence of reducing sugars contributes to the formation of carbonyl-containing glycation products (see Fig. S1), which then mature into advanced glycation end products via subsequent reaction mechanisms (*i.e*., AGEs or Maillard reaction products)^24,25^. Because these pathways are non-enzymatically driven, they can continue after death. These *post mortem* reactions may contribute significantly to tissue preservation by conferring resistance to degradation to the structural proteins that form the basis for the vessel structure. The existing biomedical and materials engineering literature shows that the accumulation of these non-enzymatic crosslinks between or within structural proteins significantly reduces their susceptibility to common degradation pathways, because as these crosslinks accumulate, vessel walls increase in stiffness^12,17,26^ and become more resistant to biological turn-over^12^ and/or enzymatic degradation^27^.

The involvement of structural proteins in Fenton chemistry and glycation crosslinking pathways yields a suite of diagnostic characters that can be detected, targeted, and characterized using a variety of techniques. For example, the metal-oxide precipitates^9^ and carbonyl (C=O)-containing crosslinks resulting from these processes (see Fig. S1), together with the formation of end product AGEs, contribute to changes in the spectroscopic properties of tissues^24^. In particular, finely crystalline iron oxide, which appears reddish-brown in colour depending on oxidation state, has been observed in the walls of ancient vessel tissues recovered from multiple specimens^9,10^, and the typical brownish hue of fossilised organic tissues has been attributed as much to AGE formation as to the presence of metal-oxide precipitates^28^.

To test our hypothesis that these early diagenetic processes could have contributed to the survival of these *T. rex* microvascular structures from deep time, we conducted an actualistic experiment in which non-enzymatic crosslinks of known character were induced in extant chicken type I collagen (recovered from the cortical bone of a chicken tibia) through Fenton and glycation pathways, using reactant concentrations relevant to known vertebrate systems (see Methods). We targeted type I collagen because it is the dominant protein in vertebrate tissues, and we derived our reference tissues from chicken because this species is phylogenetically close to non-avian dinosaurs. Moreover, this protein has been widely characterized and is readily available. Crosslink characteristics in the treated chicken tissues were directly compared with untreated tissues using synchrotron radiation Fourier-transform infrared spectroscopy (SR-FTIR) conducted in transmission mode. Then, we compared both treated and untreated states to our observations of the microvascular tissues recovered from the compact/cortical bone of the *T. rex* specimen (USNM 555000). Prior to SR-FTIR analysis, we employed optical and electron microscopy methods, small angle X-ray scattering (SAXS), and *in situ* immunohistochemistry (IHC) to test the endogeneity of these tissues. Finally, micro-focused X-ray fluorescence (*µ*-XRF) and extended X-ray absorption near-edge structure spectroscopy (*µ*-XANES) were utilized to document the presence of transition metals in the *T. rex* tissues and then to determine their chemical speciation, for iron compounds in particular, as further supporting evidence of the likely contribution of these crosslinking mechanisms to vessel tissue preservation.

## Results

### SR-FTIR analysis of crosslink character in chicken type I collagen

**Figure 1:**
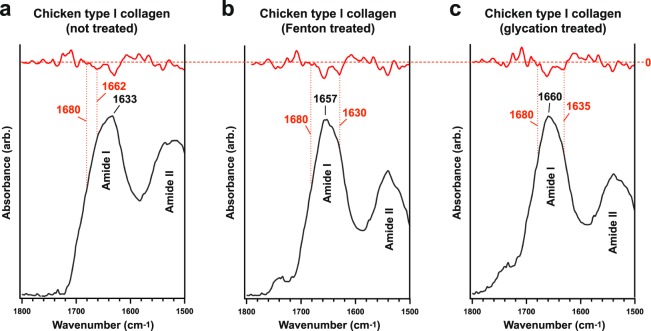
Amide I sub-band localisation of untreated and treated chicken type I collagen in SR-FTIR spectra. Sub-bands (*β*-sheet, ~1633 cm^−1^; triple-helix, ~1658–1660 cm^−1^; intermolecular, ~1683–1690 cm^−1^) are indicated in the figures. Red traces denote second derivatives of experimental curves. Although the intermolecular sub-band typically presents at lower wavenumber, the identified value was the nearest local minimum in each of the second derivative traces and consistently appears across all samples; therefore, in this sample, the intermolecular sub-band was indexed at 1697–1699 cm^−1^.

We induced crosslinks in fresh, extant chicken type I collagen using either the Fenton reagent or iron-catalysed glycation^29,30^, and then used transmission SR-FTIR to analyse each tissue. Curve fitting and second derivative analysis identified locations and relative absorption intensities of key sub-bands of the protein Amide I band, which is consistent with, but not exclusive to, protein secondary structure. The Amide I band of untreated immature type I collagen was observed at ~1633 cm^−1^ (Fig. 1a), consistent with un-crosslinked (immature) type I collagen^30–32^.

Post-treatment SR-FTIR analysis (Fig. 1b,c) revealed three significant changes to chicken fibrillar collagen profiles when compared to untreated samples. *First*, treatment with the Fenton reagent (1657 cm^−1^) and iron-catalysed glycation (1660 cm^−1^) caused an apparent blue shift in the Amide I band (Fig. 1), as the absorption of the triple-helix (~1658–1660 cm^−1^)^31,33^ and intermolecular sub-bands (~1683–1690 cm^−1^)^30,34^ increasingly dominated, consistent with changes in the secondary structure of mature collagen due to increased intramolecular crosslinking^31–33^. *Second*, both glycation and Fenton treatments gave rise to a non-peptide carbonyl band at ~1739 cm^−1^ (Fig. 1b,c). Prior studies on aged collagenous tissues^35^, glycated fibrillar collagens^25,34–36^, and demineralised tissues of Mesozoic fossils^4,9^ all reported this same spectral feature to varying relative intensities, which can result from Fenton-type reactions in the immediate vicinity of peptide sequences^36^, leading to peptide crosslinking and the subsequent formation of aldehydic carbonyls^37^, or immature, intramolecular crosslinks (see Fig. S1). *Third*, a carbohydrate band (~900–1100 cm^−1^, Fig. S2)^25,34^ developed in extant tissues after glycation. We report this latter observation simply as evidence of crosslink introduction, but explore the feature no further because the shape of this band is highly dependent on the nature and amount of different sugar molecules present during crosslinking^38^ (*i.e*., in this work, we only carried out glycation analyses with D-glucose as a reagent), and because in these chicken tissues, the intramolecular crosslinks that have formed are of the immature variety, lacking exposure to the necessary subsequent pathways to mature into intermolecular crosslinks, or AGEs^24,25^.

### Characterisation of *T. rex* vessel structures and evidence for endogenous structural proteins

**Figure 2:**
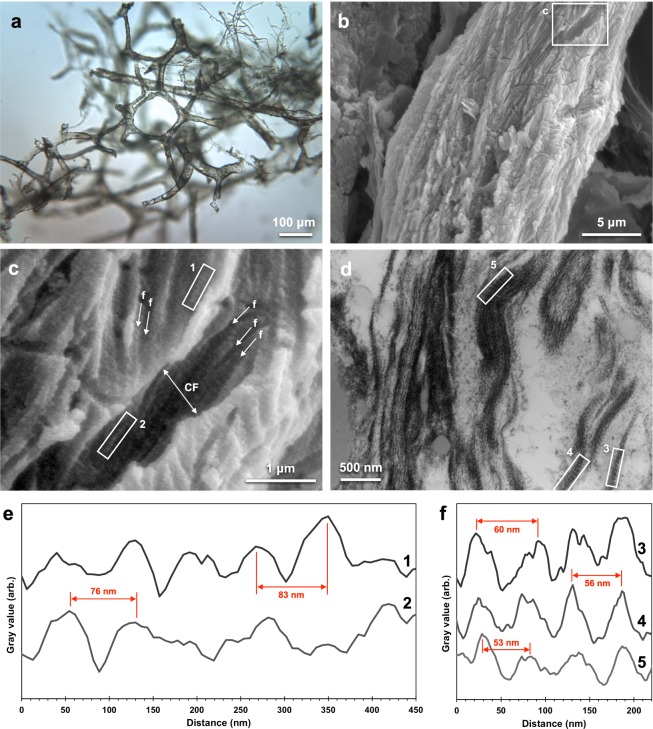
Microscopy images of *T. rex* vascular tissue and associated analysis of fibrillar collagen banding. (**a**) Transmitted VLM of *T. rex* soft tissue shows an extensive network of hollow, pliable, vascular structure and typical brown hue. (**b**) SEM image of the surface of a vessel. (**c**) Magnified image of (**b**) detailing features consistent with collagen fibre bundles (collagen fibril, “f”; collagen fibre, “CF”). Average fibril width was measured as 110 nm, and average fibre width, 1.0 *μ*m. (**d**) TEM image of fibrous features observed in a longitudinal vessel cross-section. Intensity profiles of banded texture in (**e**) boxes 1 and 2 in **c** and (**f**) boxes 3, 4, 5 in (**d**) with example peak-to-peak distances (SEM average, ~74 nm; TEM, ~56 nm) called out in red. See Fig. S6 for precise d-spacing values determined using SAXS. For comparison to a modern blood vessel network in bone, see Fig. 5b of ref.^39^.

Three types of vessels were liberated from demineralised (see Methods) *T. rex* cortical bone (Fig. S3), characterised as: (1) extensive, brown-hued, pliable vessel networks (Fig. 2a), (2) fragmented, opaque structures (Fig. S4a), and (3) fragmented, semi-translucent structures (Fig. S4a) under visible light microscopy (VLM). The pliable vessels (1) were hollow, ranged from 10–40 *µ*m in width, and demonstrated branching networks consistent in size and morphology with microvascular tissues in extant bone^39^. Energy-dispersive X-ray spectroscopy (EDS; Fig. S4c–e) coupled with scanning electron microscopy (SEM) and, separately, *µ*-XRF spectroscopy (Fig. S5) confirmed that the differences observed in VLM between the soft, transparent (1) and opaque (2) and/or semi-translucent (3) vessels arose from compositional variation between these samples. The opaque (2) vessels were dominated by iron oxide, while the semi-translucent fragments (3) were composed of barium, sulphur, and oxygen. We focused all further analyses on the pliable (1) vessel networks, because they appeared most similar to those observed in extant bone tissue, and thus were presumably less altered.

When analysed by SEM, the pliable *T. rex* vessels (Fig. 2b) exhibited ordered fibrous structures across their outermost surface. At higher magnification (Fig. 2c), the vessel exteriors were roughly textured and finely striated, exhibiting features morphologically consistent with fibrillar collagen^13^. These same striated, fibrous features were also observed (Fig. 2d) in longitudinal sections of vessels imaged by transmission electron microscopy (TEM; see Methods). The average structural features included a fibril width of 110 nm, fibre bundle width of 1.0 *µ*m, and ~74-nm periodicity (d-spacing) of fine striations as recorded in SEM (Fig. 2e) and 56 nm, in TEM (Fig. 2f). We used SAXS to determine the periodicity of *T. rex* fibrillar structures more precisely, calculating a d-spacing of 66.5 nm (Fig. S6).

**Figure 3:**
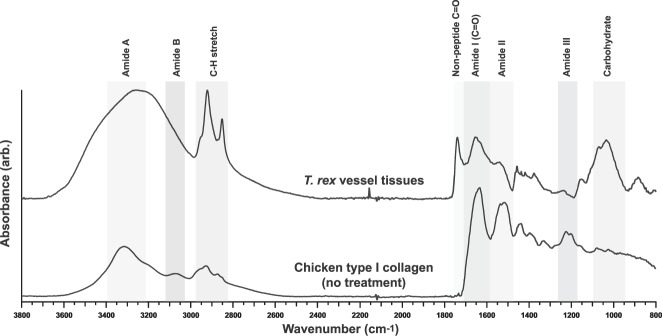
SR-FTIR full spectra of isolated *T. rex* vascular tissue and chicken type I collagen (no treatment). All key bands for the identification of protein (Amide I, Amide II, Amide III) are present in the dinosaur tissue spectrum. The *T. rex* spectrum also presents a strong non-peptide carbonyl (C=O) band at 1739 cm^−1^ and a carbohydrate band at ~1010 cm^−1^.

These combined features are consistent with those seen in extant vessels liberated from cortical bone (see Fig. 3 of reference^1^), and with fibrillar collagen (predominantly type I in the tunica media and tunica externa of blood vessels^11,12,16^), which shows striations with an accepted d-spacing of 67 nm^40^. Similar features have been observed in vessels^1^ and mineralised fibrils^8^ recovered from other fossil specimens. Importantly, these vessel networks are too large, and not consistent in morphology, with fungal hyphae^41^.

The SR-FTIR spectrum of the *T. rex* vessels was dominated by Amide I and Amide II bands, at values consistent with the absorption bands of extant chicken type I collagen (Fig. 3)^36,42,43^. The maximum absorption intensity of the Amide I band, corresponding to carbonyl (C=O) stretching, was located at ~1657 cm^−1^; the Amide II band (N-H bending), at ~1541 cm^−1^; the CH_2_ bending vibration also seen in collagen, at ~1458 cm^−1^; and the C-OH stretching vibrations of carbohydrate moieties (associated with AGE formation)^25,34^ at ~1036 and ~1066 cm^−1^ were observed in both the treated extant and ancient tissues. Specifically, the location of the Amide I band for the dinosaur tissue is indicative of a predominant *α*-helix structure^31^, consistent with the triple *α*-helix structure of mature (crosslinked) fibrillar collagen. The C-H stretch (2852 and 2922 cm^−1^) and non-peptide carbonyl (1739 cm^−1^) bands can be associated with the presence of lipids and/or proteins^25^ and are discussed in more detail below. Because this analysis was carried out in transmission mode, all layers of the vessel structures (including the layers exhibiting morphological characters consistent with fibrillar collagen; see Fig. 2b,c) were probed during analysis.

### Immunohistochemistry (IHC) of *T. rex* vessels

The above results indicate that these *T. rex* tissues are composed of a significant fraction of protein-derived compounds, and possess a degree of structural character consistent with fibrillar collagen. As a corroborating line of evidence, we also conducted *in situ* IHC analyses to identify and localise to the micron-scale protein-specific epitopes of the structural proteins elastin and type I collagen, and other proteins associated with extant microvascular tissue (actin, tropomyosin, haemoglobin). IHC was selected for identification of protein constituents because of the specificity of the vertebrate immune response, which can be employed to differentiate endogenous proteins associated with blood vessels^44^ from those arising due to exogenous contaminates^45^. Specifically, actin proteins constitute the cytoskeleton of blood vessel endothelial cells^46^ and contribute to the functionality of the thin filaments, along with tropomyosin, in smooth muscle cells^47^. Tropomyosin is a component of muscle tissues and is localised within the tunica media of vessel walls, where smooth muscle cells reside^48^. Thus, tropomyosin, actin, and collagen often co-localise in extant vessels at the lower magnifications represented herein. Elastin is a major component of the basement membrane that forms the boundary (*i.e*., internal elastic lamina) between the endothelial cells of the tunica intima and the collagen and smooth muscle cells forming the tunica media^48^. Haemoglobin, the major protein within vertebrate erythrocytes (red blood cells), is abundant in highly vascularised bone. When released during *post mortem* haemolysis, haemoglobin protein can interact with and adsorb onto surrounding tissues; in modern vertebrates, haemoglobin imparts a red hue to bone and the walls of blood vessels^10^. Thus, these proteins constitute a reasonable suite of targets for IHC techniques, and their *in situ* identification in fossil blood vessels supports their endogeneity. Concurrently, we tested for reactivity of these vessels to antibodies raised against peptidoglycan, a component of bacterial biofilms, and employed established negative controls to account for non-specific reactivity.

**Figure 4:**
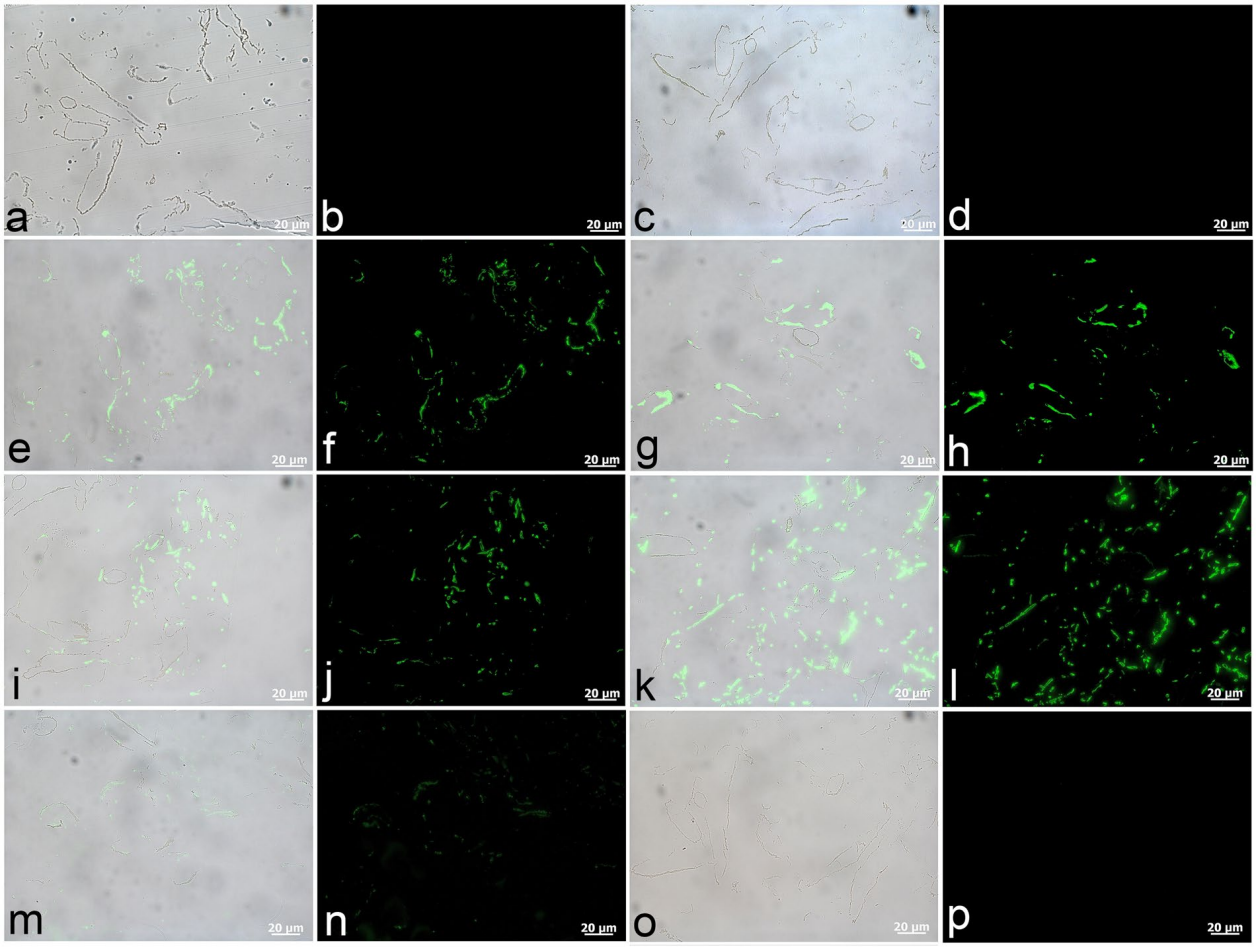
*T. rex* tissues exhibit positive antibody binding to protein components of extant vascular tissue. (**a,c,e,g,i,k,m,o**) Are composite images in which fluorescence corresponding to antibody-antigen complexes is overlain upon VLM images of vessel sections, with adjacent images (**b,d,f,h,j,l,n,p**) captured using a fluorescent filter. (**a–d**) No spurious binding was observed for negative controls in which vessels were exposed to secondary antibodies raised against the host species of all other antibodies used, *i.e*., mouse (**a,b**) and rabbit (**c,d**). (**e,f**) Positive binding of dinosaur vessels to actin antibodies can be seen in thin, evenly distributed layers, and (**g,h**) more broadly distributed binding is apparent for muscle tropomyosin antibodies. Antibodies to both (**i,j**) type I collagen and (**k,l**) elastin bind positively to these *T. rex* vessels. (**m,n**) Antibodies raised against ostrich haemoglobin exhibit comparatively lower binding intensity. (**o,p**) No reactivity of dinosaur vessels to antibodies against bacterial peptidoglycan was observed.

As shown in Fig. 4, antibodies raised against all of the components of extant vasculature that we tested exhibited positive binding to the dinosaur vessel walls. Composite images (Fig. 4a,c,e,g,i,k,m,o) were created by overlaying fluorescence images corresponding to antibody-antigen complexes upon VLM images of vessel sections. A fluorescent filter was used during capture of the adjacent images (Fig. 4b,d,f,h,j,l,n,p), which show the localisation and distribution of antibody-antigen complexes (green fluorescence). Negative controls to account for non-specific binding (Fig. 4a–d) were conducted by exposing vessels to secondary antibodies raised against mouse (a,b) and rabbit (c,d), the host species for antibodies employed. No signal was observed, indicating that spurious binding of the secondary antibodies to the tissues was not responsible for positive signal in the images. The response of these dinosaur vessels to actin antibodies (Fig. 4e,f) was observed as a thin and evenly distributed layer, whereas antibodies raised against the muscle protein tropomyosin (Fig. 4g,h) were bound with greater intensity over a broader portion of the vessel walls, consistent with the distribution of these proteins in extant vessels^46,47^. The dinosaur vessels also reacted to type I collagen antibodies (Fig. 4i,j), although elastin antibodies were bound with greater intensity (see Fig. 4k,l). These two proteins are highly evolutionarily conserved in certain regions of their respective sequences^49,50^, making them good targets for fossil studies. Antibodies raised against ostrich haemoglobin^10^ also showed positive binding, although at comparatively lower intensity (Fig. 4m,n). No reactivity was seen for vessels exposed to antibodies against bacterial peptidoglycan (Fig. 4o,p), eliminating the possibility that these structures arose from microbial contamination^45^.

### Investigation of possible crosslinking mechanisms in *T. rex* vessel structures

We previously demonstrated that the treatment of extant microvascular tissue with haemoglobin, an Fe-coordinating protein, can significantly enhance stability over multi-year time frames^10^, in effect acting as a preserving agent. Here, we extend this experimental observation to propose that enhanced resistance to degradation is due in part to Fe-catalysed non-enzymatic crosslinking of molecules comprising structural tissues, with haemoglobin suggested as the primary source of such Fe in vessels undergoing diagenesis.

To test whether *post mortem* structural protein crosslinking (specifically, on type I collagen) could have enhanced the resistance of these *T. rex* vessel structures to degradation/diagenetic alteration processes, we employed two experimental approaches: for our first approach, we analysed the spectral features of the dinosaur tissues with the same approach applied to chicken type I collagen, and found that all three significant changes observed in the treated (crosslink-induced) chicken tissues were, to some degree, identifiable in the dinosaur vessel structures. In this work, our focus was specifically on fibrillar collagen (predominantly type I collagen in extant vessel tissues), for several reasons: (1) this structural protein type imparts structural integrity to modern vessels, (2) fibrillar collagen is most abundant in the tunica media and tunica externa, with the potential to encapsulate and protect the innermost contents of fossil vessel networks, and (3) our SEM and TEM imaging studies of these *T. rex* vessels clearly demonstrated extensive fibrillar surface features on the outermost preserved layers of the vessels as well as in their longitudinal sections.

**Figure 5:**
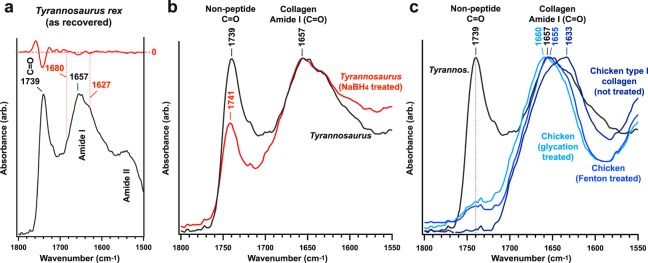
SR-FTIR analysis of *T. rex* vascular tissue, NaBH_4_ reduced *T. rex* vascular tissue, chicken type I collagen without treatment, and chicken type I collagen treated with Fenton reagent and iron-catalysed glycation. (**a,b**) Average FTIR spectra in the non-peptide carbonyl and protein amide I regions for all five samples. (**a**) Significant reduction in the non-peptide carbonyl band follows treatment of *T. rex* vascular tissue with NaBH_4_, which reduces (immature) peptide crosslinks. The blue-shifted Amide I band of the dinosaur tissue, Fenton reagent-treated chicken type I collagen, and Fe-catalysed glycation-treated chicken type I collagen indicate increasing *α*-helix structure (~1660 cm^−1^) as the higher-energy triple-helix and intermolecular sub-bands (see Fig. 1 for method of identification) increasingly predominate the spectra. The development of aldehydic carbonyl, ketoaldehyde, and/or immature ketoimine bands in both treated chicken tissues is consistent with the strong carbonyl band in the dinosaur tissue.

Using transmission SR-FTIR, the maximum absorption intensity of the Amide I band of the *T. rex* tissue was observed at 1657 cm^−1^ (Fig. 5a). Sub-band analysis (Fig. 5a) indicated that this band location was due to increased relative intensities of the triple-helix (~1658–1660 cm^−1^)^31,33^ and intermolecular (~1683–1690 cm^−1^)^33,34^ sub-bands, as observed in both samples of crosslinked chicken vessels. The maximum absorption intensity of the *T. rex* Amide I band fell between the Amide I bands recorded for chicken type I collagen crosslinked using Fenton chemistry (1657 cm^−1^) and Fe-catalysed glycation (1660 cm^−1^), suggesting that both mechanisms could have contributed to *post mortem* crosslinking in this tissue.

The prominent non-peptide carbonyl band (Fig. 5b) observed in the dinosaur tissues (1739 cm^−1^) also developed in the chicken type I collagen tissues treated by both crosslinking mechanisms, because non-peptide carbonyls were introduced within immature, intramolecular crosslink structures^25,34–36^. This same spectral feature has been recorded, but not discussed, in two other works on ancient fossil soft tissues^4,9^. Concurrently, crosslinking of the chicken tissues enhanced the absorption intensity and prominence of C-H stretch bands, representing aliphatic protein groups at 2850 and 2930 cm^−1^ (Fig. 1). These same bands exhibited strong absorption in the dinosaur tissues (Fig. 3). Although C-H stretch and non-peptide carbonyl (C=O) bands can also be associated with lipids, the presence of lipids is only supported if strong and sharp (cusp-like) absorption bands associated with C-H bending modes are present in the fingerprint region (~1450 cm^−1^) and the absorption feature of olefins^51^ is present at ~3010 cm^−1^. In contrast, protein methyl group absorption in the region of (1500–1300 cm^−1^) exhibits a broader, more-rounded character (*e.g*., chicken spectrum in Fig. 3). Because the spectrum for the *T. rex* tissue exhibits neither strong, sharp C-H bending modes nor strong olefin absorption, we associated the C-H stretch and non-peptide carbonyl bands observed in the dinosaur tissue with the presence of protein structures.

The *T. rex* SR-FTIR spectrum also exhibits increased absorption in the carbohydrate region. The shape of the absorption band in this region is dependent on the nature and amount of different sugar molecules present during crosslinking^38^. In this specimen, the doublet presenting at ~1036 and ~1066 cm^−1^ (Fig. 3) is highly consistent in energy position, absorption intensity, and overall feature shape with observations of AGE ‘hotspots’ formed in the cardiac tissue of mice fed high-glycemic-index diets (specifically, amylopectin, which is a glucose starch)^25^. Increased absorption intensity in this same region was observed in both the Fenton chemistry- and glycation-treated chicken type I collagen samples, suggesting that the absorption band in the *T. rex* tissue could be due to multiple effects. Although a degree of contribution from enzymatic glycosylation^52^ cannot be ruled out, the combination of features present, in coordination with the carbohydrate band absorption shape, strongly indicate that this feature arises from non-enzymatic crosslinking mechanisms. Furthermore, the differences between the carbohydrate band of *T. rex* and that of either crosslinked chicken sample are likely due to a difference in crosslink state: the immature, intramolecular crosslinks induced in the chicken tissues lacked exposure to the subsequent pathways necessary for maturation into mature, intermolecular crosslinks (AGEs).

For our second experimental approach, we treated bulk *T. rex* tissue (Fig. 5c) with NaBH_4_. This compound reduces the carbonyl group within immature crosslinks, thereby decreasing the non-peptide carbonyl band absorption intensity (see Fig. S1)^53^. After treatment, we observed significantly lower intensity of the non-peptide carbonyl band at ~1739 cm^−1^ in the *T. rex* tissues, suggesting that a significant fraction of the crosslinks in this tissue are immature. The data suggest that this *T. rex* tissue possesses both intramolecular and intermolecular crosslink types.

**Figure 6:**
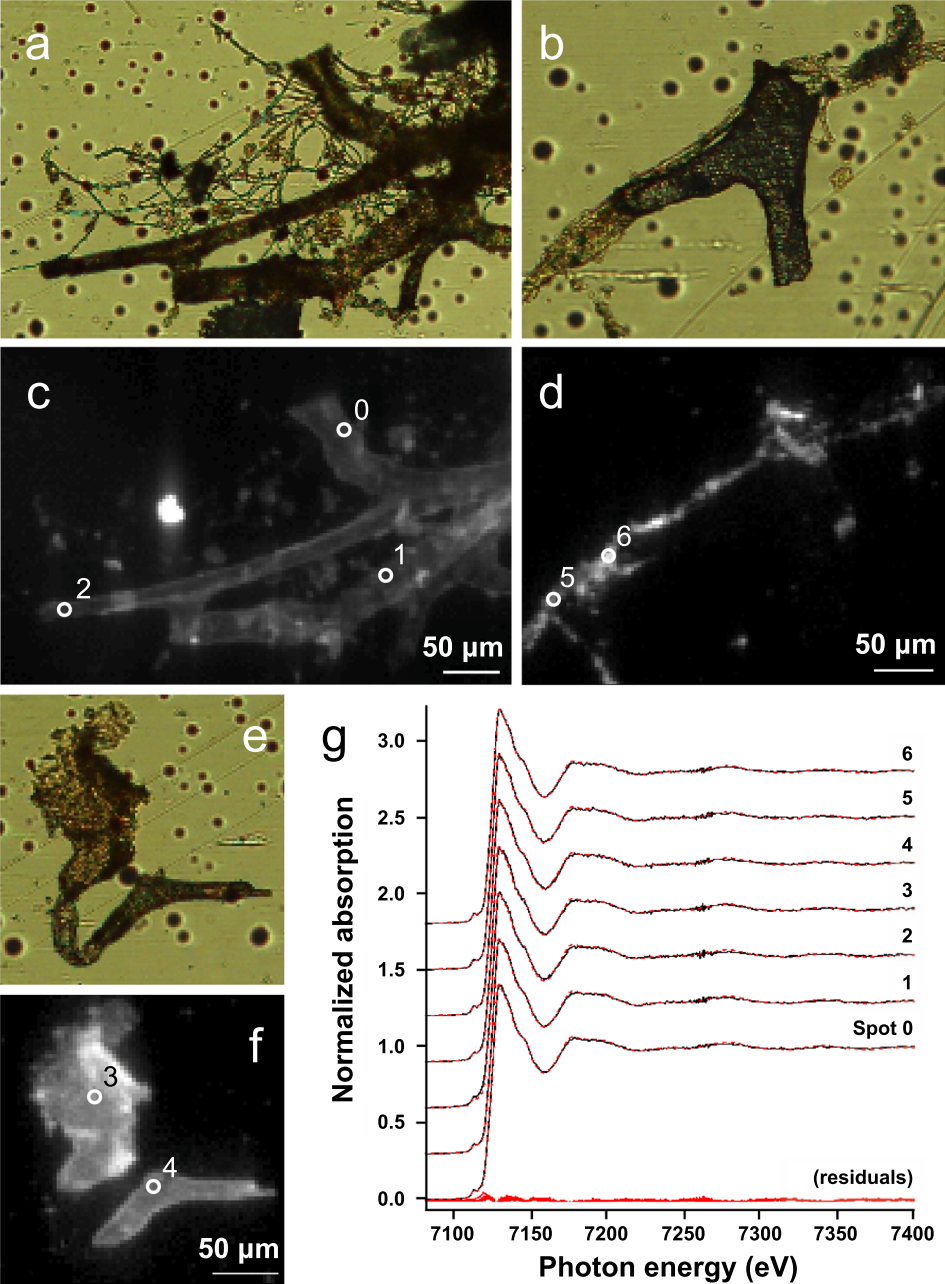
X-ray microprobe analysis of iron in the *T. rex* vascular tissues. (**a,b,e**) Optical microscope images of vessel tissues and (**c,d,f**) corresponding iron *μ*-XRF distribution maps recorded at 10 keV. Brighter pixels correspond to higher Fe content. All scale bars are 50 *μ*m. Additional elemental maps of regions (**a**) and (**b**) can be found in Fig. S5. In (**b,d**) the vessel structure is not an organic tissue but a mineralised cast rich in Ba and S (see Fig. S5). Such fine-scale variation in preservation underscores the notion that preservation depends on the microenvironment. Numbered white circles indicate locations of Fe *μ*-XANES analysis. (**g**) Stacked normalised Fe K-edge extended XANES spectra of spots 0–6. Fits are shown in red dashed lines, with corresponding residuals plotted at the bottom. All spectra match to goethite (*α*-FeO(OH)) with normalised sum-square values ranging from 0.59 to 1.93·10^−4^. For comparison, an example set of the iron bearing reference spectra used are displayed in Fig. S7.

Elemental mapping using *µ*-XRF (Fig. 6a,b,e) revealed that Fe was the only metal concentrated within the dinosaur vessel tissues (Figs. 6c,d,f and S5). Extended *µ*-XANES at the Fe K-edge on seven tissue locations (Fig. 6g) showed Fe^3+^ embedded in the vessel walls. These spectra, which were recorded up to 300 eV past the K-edge into the extended X-ray absorption fine structure (EXAFS) region, were used to identify the nature of Fe oxides present by simultaneously fitting several of the distinctive EXAFS features (see Fig. S7). Least-square linear combination fitting of the Fe K-edge XANES spectra, using a published spectral database of >90 Fe standard compounds, showed that these vessels contained finely crystalline goethite (*α*-FeO(OH)), a mineral we previously observed in vascular tissues recovered from two different dinosaur specimens^10^.

## Discussion

The identification of still-soft tissues and cellular structures in a suite of Mesozoic fossils^1–5,8,10^, and claims of endogenous proteins preserved within these materials^3,4,8–10,28^, is controversial because it challenges both conventional wisdom and theoretical kinetics, which preclude the persistence of proteins over geological time scales^54,55^. Data supporting endogeneity have been viewed with scepticism, in part because no mechanisms have been identified that could reasonably contribute to such preservation.

Our data support the presence of vertebrate-specific endogenous proteins, localized to these soft tissue dinosaur structures, including the presence of a significant quantity of type I collagen, consistent in location and chemistry with the vasculature of extant vertebrates^11,12,16^. Furthermore, we provide data to support a two-step mechanism that stabilises biomolecules and vessel structures shortly after organismal death, promoting their persistence within densely mineralised skeletal elements.

When biological iron, which exists in the thermodynamically unstable Fe^2+^ state, is released from complexing molecules, it oxidises rapidly, generating free radicals^10,56–58^ in the conversion from Fe^2+^ to the more stable and insoluble Fe^3+^ form. We hypothesise that early in diagenesis, perhaps immediately *post mortem*, iron-mediated Fenton and glycation pathways contributed to enhanced *T. rex* tissue longevity by rapidly crosslinking arginine and lysine residues of elastin and fibrillar collagen (and other vascular proteins) within and surrounding the blood vessels. This Fenton-type reaction would have been heavily favoured by conditions of oxidative stress as Fe-containing haemoglobin was liberated into and degraded within the vessel lumen during haemolysis^56–59^. The iron would have first bound to a metal-binding site on collagen, normally occupied by Ca, producing hydroxyl radicals that would then have instantaneously reacted with local amine groups^10^, leading to aldol condensation and crosslinking among adjacent protein molecules. Alternatively, or perhaps simultaneously, autoxidised carbohydrates in non-enzymatic glycation would have undergone condensation with the terminal amine groups of lysine, leading to ketoaldehyde formation and protein crosslinking. Both processes are catalysed by transition metal species, including iron^60^, and hence would have driven crosslinking in the immediate vicinity of the Fe catalyst^10^. Hence, the central role of Fe in structural protein crosslinking also provides a causal mechanism to explain the observations of intimately associated and distributed iron oxyhydroxides within the dinosaur tissues reported here. This idea is fully supported by the formation of iron oxyhydroxide precipitates observed in the walls of extant vascular tissues incubated in haemoglobin, and ancient vascular tissues recovered from other dinosaur specimens^10^.

Overall, the role of iron in generating free radicals is central to both the Fenton reaction and transition metal-catalysed glycation, and the immature, intramolecular crosslinks resulting from both pathways exhibit non-peptide carbonyl functional groups, which have been recorded, although not commented on, in other spectral analyses of ancient tissues^4,9^. We show that the chemical complexity of crosslinks in the dinosaur structural proteins varies widely, with the presence of both mature, intermolecular crosslinks identifiable *via* SR-FTIR Amide I sub-band analysis^31–33^ and based on the shape of the carbohydrate absorption band in the *T. rex* tissue (consistent with AGEs)^25^ and immature crosslinks verified by treatment with the reducing agent NaBH_4_^53^. Although lipid peroxidation can also give rise to a carbonyl band at the same energy as the non-peptide carbonyl band resulting from intramolecular crosslinking, the full FTIR profile of a lipid is distinct from that of a protein. The *T. rex* tissues were found to exhibit a predominantly proteinaceous character, strongly indicating that the majority of the carbonyl groups in this sample derive from crosslinks associated with protein compounds. Molecular crosslinks (essentially, hyper-crosslinking) would have afforded exceptional resistance to mechanical, biological, and thermal degradation^12,17,26,27,61–64^. Moreover, *post mortem* lipid peroxidation events would have resulted in cell membrane degradation^65^, contributing to increased diffusion of serum proteins (*e.g*., haemoglobin) and myoglobin-containing Fe in smooth muscle cells to the structural components of vessel walls during short-term diagenesis. In contrast, lipoprotein oxidation events associated with ROS-mediated mechanisms could lead to the uptake of these compounds into vessel walls; therefore, it would not be unreasonable to expect the observation of lipoprotein plaque deposition in other fossil soft tissues, similar to atherosclerotic plaque formation in living systems^65^.

The extensive presence of finely crystalline goethite (*α*-FeO(OH)) in these fossil vessels suggests that conditions favoured thermodynamically-driven nucleation over kinetically-driven growth, and that this mineral formed on a large number of distributed nucleation sites (*i.e*., Ca-binding sites), as previously demonstrated^9^. In accordance with the fundamentals of mass transfer phenomena, diffusion mechanisms are aided by factors such as small crystallite size (large surface area), elevated temperature, solubility (K_sp_), pH, and/or long time periods. Ultimately, growth within the sub-microscopic features of the microvascular walls would have intrinsically restricted crystallite size.

Goethite formation from aqueous ferrihydrite (Fe^3+^) significantly predominates over haematite in the range from pH 4 (>95% goethite) to pH 5 (>80% goethite)^66^, with the highest crystallinity of goethite exhibited at pH 4^66^. Goethite solubility at neutral pH is extraordinarily low^66^. In this work and in our previous study^10^, we observed the presence of finely crystalline goethite throughout the fossil blood vessels investigated, suggesting that the mineral formed under acidic pH, which is consistent with the development of a hypoxic *post mortem* environment^67^. Over time, the vessel cavity pH would have been regulated primarily by ground water and the strong buffering capacity of bone mineral^67,68^, maintaining values near neutral pH and, as a result, preventing goethite dissolution. Micromobilisation of apatite crystals from the encapsulating bone onto the vessel walls could have provided a secondary form of stabilisation via encapsulation (note that such crystals would have been removed by the demineralisation process used to liberate the *T. rex* vessels in this work, and hence would not have been observed during microscopy studies). Therefore, we argue that such formation conditions are consistent with the Fe-catalysed crosslinking mechanisms tested in this work, and not of exogenous, geological origin.

We provide additional support for our hypothesis by demonstrating that both chemical pathways tested herein were capable of inducing crosslink formation in chicken type I collagen tissues within 48 h at reactant concentrations consistent with anticipated values in dinosaur blood systems (see Methods). Such observations reinforce the likely role of Fenton- and glycation-type transition metal (Fe)-catalysed pathways in the early and rapid diagenetic stabilisation of microvascular tissues in the burial environment. As we have previously shown, Fe in haemoglobin compounds can significantly enhance microvascular tissue stability over multi-year time frames^10^.

## Conclusions

These data represent the first comprehensive chemical and molecular characterisation of vascular tissues recovered from this *T. rex* specimen (USNM 555000). By combining synchrotron and laboratory techniques with verified and well-understood immunological, diffraction, and microscope imaging methods, we provide the first identification of reducible, intramolecular (immature) and irreducible, intermolecular (mature) crosslinks in preserved, ancient vessel tissues. These data strongly support the previous hypothesis^10^ invoking transition metal (Fe)-mediated mechanisms as an agent of vessel preservation. Exposure of extant chicken type I collagen tissues to Fenton chemistry and transition metal-catalysed glycation rapidly induces chemical modifications also observed in the dinosaur tissues studied here.

In addition to these inherent molecular features conferring resistance to degradation in tissues that possess them, the sequestration of proteinaceous components within mineralised tissue has also been demonstrated to promote longevity, by restricting degradative pathways in the immediate and long-term *post mortem* environment^69,70^. We hypothesize that the enzymatic and non-enzymatic pathways described herein, coupled with adsorbance to the mineralized components of bone, can result in exceptional preservation of the original organic components of dinosaurian vascular tissues.

We have shown that actualistic taphonomy provides mechanisms for preserving endogenous soft tissues previously considered impossible, that these mechanisms provide a means for preserving constituent molecules to the degree that they may shed light on evolutionary relationships, and that certain aspects of the immediate microenvironments of degradation can be deduced by examining the chemistry of preservation. These results confirm earlier findings^1–3,7^, and those reported in other studies^4,8^, and shed light on the possible suite of processes involved in fossilisation at the molecular level. The ability to localize structural proteins within vascular tissues and correlate these observations to chemical and structural alterations in fossil soft tissues will contribute to the development of a comprehensive model of mechanisms that contribute to vascular tissue survival from deep time.

## Methods

For a detailed description of the methods and techniques used, please see the Supplementary Information.

## Supplementary information

Mechanisms of soft tissue and protein preservation: Supplementary Information
