# Supplementary material for: Mechanisms of soft tissue and protein preservation in Tyrannosaurus rex
Source: Sci Rep. 2019 Oct 30;9:15678. doi: 10.1038/s41598-019-51680-1 (PMC6821828; doi:10.1038/s41598-019-51680-1)
Supplement: Supplementary file 1 — Mechanisms of soft tissue and protein preservation: Supplementary Information [file 41598_2019_51680_MOESM1_ESM.docx]

**Mechanisms of soft tissue and protein preservation**

**in *Tyrannosaurus rex***

**Elizabeth M. Boatman^1*^, Mark B. Goodwin^2^, Hoi-Ying N. Holman^3^, Sirine Fakra^3^, Wenxia Zheng^4^, Ronald Gronsky^5^, Mary H. Schweitzer^4,6,7,8^**

^1^Department of Engineering, Wake Forest University, Winston Salem NC 27101, USA

^2^Museum of Paleontology, University of California, Berkeley CA 94720, USA

^3^Advanced Light Source, Lawrence Berkeley National Laboratory, Berkeley CA 94720, USA

^4^Department of Biological Sciences, North Carolina State University, Raleigh NC 27695, USA

^5^Department of Materials Science and Engineering, University of California, Berkeley CA 94720, USA

^6^Department of Geology, Lund University, Lund, Sweden

^7^North Carolina Museum of Natural Sciences, Raleigh NC 27601, USA

^8^Museum of the Rockies, Montana State University, Bozeman MT 59715, USA

**SUPPLEMENTAL INFORMATION**

***Materials and methods***

**Sample preparation.** Diaphysis portions of *Tyrannosaurus rex* (USNM 555000) and freshly recovered (less than 10 hours *post mortem*) modern chicken tibia bones were wafer-sectioned using diamond-coated blades. The sections were then demineralised in 0.5 M EDTA pH 8.0 (Fisher Scientific) at room temperature for 2-3 weeks. After demineralisation, specimens were flushed with ultrapure distilled water and refrigerated at 6$^{\circ}$C until use. Dedicated facilities and equipment were used where possible; when not possible, sterile methods were employed, and *T. rex* tissues were processed first to ensure that no contamination of the fossil tissue by modern tissue could occur. In particular, samples intended for *in situ* immunohistochemistry (IHC) were prepared in a laboratory designated to analyses of fossil tissues.

***In situ* IHC.** Demineralised vessels were incubated with iron chelator pyridoxal isonicotinoyl hydrazone (PIH) solution^1^ (5 mM PIH in 50 mM NaOH) overnight at room temperature, then washed with PBS buffer. The PIH was synthesised according to a previously reported protocol^2^. In short, 2.5 mMol of pyridoxal in water was mixed with equimolar amounts of 2.5 mMol isonicotinic acid hydrazine (in a small amount of water, for a nearly saturated solution), heated for approximately 5 min in a water bath at 100$^{\circ}$C, allowed to cool, and then dried under vacuum.

After PIH treatment, dinosaur vessels were then partially dehydrated in 70% ethanol twice for 20 min each, then infiltrated with a 2:1 mixture of LR White (EMS cat# 14383, hard grade) and 70% ethanol for one h. Samples were then incubated in 3 changes of undiluted LR White for 1 h each time. Specimens were placed in gelatin capsules (EMS cat# 70100), covered with LR White embedding medium, and polymerised at 60$^{\circ}$C for 24 h. After polymerisation, 200-nm sections were taken using a Leica EM UC6 *Ultra*-microtome and then were mounted on 6-well Teflon-printed slides (EMS cat# 63423-08) and dried overnight at 42$^{\circ}$C.

Sections were etched with 25 *μ*g/mL Proteinase K (Roche 03115879001) in PBS at 37$^{\circ}$C for 15 min, followed by 0.5 M EDTA pH 8.0 (3 incubations by 10 min each) and then NaBH_4_ (2 by 10 min). Incubations were separated by two 5-min washes in PBS. Following these steps to accomplish epitope retrieval and quenching of autofluorescence, 4% normal goat serum (NGS) in PBS was applied to occupy non-specific binding sites and prevent spurious binding. Sections were then incubated in one of the following primary antibodies: polyclonal rabbit anti-chicken actin 1:75 (Capralogics, Inc. P00851), polyclonal rabbit anti-tropomyosin 1:40 (AbCam ab11190), polyclonal rabbit anti-bovine elastin 1:75 (courtesy of R. Mecham), polyclonal rabbit anti-chicken collagen I 1:75 (USBiological C7510-13B), polyclonal rabbit anti-ostrich hemoglobin (GernScript 70594-1), or monoclonal mouse anti-peptidoglycan 1:75 (AbD Serotect 7263-1006). In each case, the respective antibody was diluted in primary dilution buffer composed of 1% bovine serum albumin (BSA, Fisher Scientific BP1660-100), 0.1% cold fish skin gelatin (Sigma G7765), 0.05% sodium azide (Sigma S-8032), and 0.01 M PBS pH 7.2; incubation was allowed overnight at 4$^{\circ}$C. To test the specificity of the chicken actin antibody, rabbit anti-chicken actin was incubated with excess chicken actin protein (Cytoskeleton cat# AS99, 10 mg/mL) at 4$^{\circ}$C overnight; the inhibited antibody was applied to the sections as above. All sections were first incubated for 2 h at room temperature with secondary antibody (biotinylated goat anti-rabbit IgG (H+L) (Vector BA-1000) diluted 1:500 in PBS for rabbit primary antibodies, biotinylated goat anti-mouse IgG (H+L) (Vector BA-9200) diluted 1:500 in PBS for monoclonal mouse anti-peptidoglycan) and then for 1 h at room temperature in Fluorescein Avidin D (FITC, Vector Laboratories A-2001). All incubations were separated by sequential washes (2 washes for 10 min each) in PBS with Tween 20 followed by two 10-min rinses in PBS. Finally, sections were mounted with Vectashield H-1000 mounting media, and coverslips were applied. Sections were examined with a Zeiss Axioskop 2 plus biological microscope and captured using an AxioCam MRc 5 (Zeiss) with 10$\times$ ocular and Plan Apochromat 63$\times$/1.4 lenses in the Axiovision software package (version 4.7.0.0). Immunofluorescence images were acquired for 100 ms. Brightness was set to -0.49, contrast was set to 1.10, and gamma was set to 0.66; these values were held constant across all samples and are relevant to all images shown in this work.

**Scanning electron microscopy (SEM) and energy-dispersive X-ray spectrometry (EDS) analyses.** Samples imaged by scanning electron microscopy (Hitachi S-4300 SE/N) were mounted with conductive carbon tape and then sputter coated with a gold target (Cressington Sputter Coater 108 auto). Images of vessel surfaces were then collected in secondary electron mode at various magnifications and an accelerating voltage of 15 kV. Energy-dispersive X-ray spectrometry (0-12 keV) was used to determine the composition of the dark and white vessel cast fragments (Thermo Electron Corp.).

**Transmission electron microscopy (TEM) sample preparation and imaging.** Demineralised, brown-hued, pliable *T. rex* vessel networks were first fixed for 48 h at 6$^{\circ}$C in 1.5% glutaraldehyde (Sigma-Aldrich, for electron microscopy use) buffered at pH 7.0 with sodium cacodylate (Electron Microscopy Sciences) according to standard procedure (Sigma-Aldrich). Fixed vessels were then dehydrated in an ethanol series (30, 50, 75, 100%) and embedded in “Spurr’s” low-viscosity resin according to the manufacturer’s recommended procedure (Ted Pella, <https://www.tedpella.com/technote_html/18300-4221%20TN.pdf>); resin blocks were cured at 70$^{\circ}$C by microwave, trimmed, and then sectioned using a diamond knife and a LKB Ultratome NOVA; ultrathin sections of embedded dinosaur vessels were then collected on 200-mesh Formvar/carbon-coated copper grids and contrast stained with uranyl acetate (Ted Pella) and lead citrate (Electron Microscopy Sciences) according to typical protocol^3^. Sections were analysed on an FEI Technai BioTwin at 120 keV accelerating voltage and multiple magnifications. Banding patterns typical of fibrillar collagen were further analysed using ImageJ by calibrating the image and then collecting grey value intensity profiles.

**Synchrotron small-angle X-ray scattering (SAXS).** Synchrotron small-angle X-ray scattering (SAXS, Advanced Light Source, beamline 7.3.3) was used to determine the d-spacing of fibrillar collagen in both the demineralised dinosaur and demineralised chicken tissues, without further treatment. Demineralised sections of each tissue were mounted as-prepared on high-quality mica windows for analysis (specimens typically dried in air within several minutes, no embedding or subsequent processing was utilized). For each specimen, the spectra recorded following multiple exposure times were averaged. Two-dimensional whole intensity patterns were processed according to standard procedures described elsewhere^4^, using silver behenate as the calibration standard. Patterns were radially integrated using the custom software macro NIKA^5^, and the resulting profiles were reported in standard Q-value (Å^-1^) versus intensity format. Plots were subsequently indexed for peak locations using curve-processing techniques and the software Igor Pro (Wavemetrics), following procedures described elsewhere^4^. (Note that the SAXS data for *T. rex* and chicken tissues were recorded on different dates; between these dates, the beam blocker and CCD were upgraded. Because all other sample preparation and data processing methods were identical, the effects of these beamline upgrades are interpreted as negligible.)

**Synchrotron Fourier-transform infrared spectromicroscopy (SR-FTIR).** SR-FTIR analyses were performed at the Berkeley Synchrotron Infrared Structural Biology (BSISB) facility (<http://bsisb.lvl.gov/>) of the ALS. Data were recorded in transmission mode on untreated and experimental sets of samples. To induce crosslinking in chicken type I collagen *via* Fenton reagent and iron-catalysed glycation, respectively, portions of demineralised tissue were incubated at room temperature for 48 h in either 10 mM aqueous FeCl_2_ (Sigma), 30 mM H_2_O_2_ (Fluka Analytical), and 100 mM phosphate buffered saline (Fluka Analytical) at pH 7.3 or with the addition of 30 mM dextrose (Sigma). Full spectra (3800-800 cm^-1^) were recorded with a 4 cm^-1^ spectral resolution in mapping mode, and 20 locations per sample were averaged to obtain the spectra reported in this work. Key peaks of interest were identified from the averaged spectra. Sub-bands of the Amide I peaks were located using the Savitzky-Golay second derivative approach with 11 points and a 3^rd^ order polynomial.

Although the concentrations of iron and sugar in *T. rex* blood cannot be known definitively, reasonable assumptions can be made based on modern vertebrate systems. The hemoglobin content of the blood of Crocodylia is ~8 g%^6^, thus the concentration of iron in *T. rex* blood would have been ~5 mM, assuming a mass fraction of iron in hemoglobin of ~3.5 mg/g, based on values for human^7^. For comparison, in our experiments, we used 10 mM FeCl_2_. Glucose levels in healthy, captive adult crocodile have been recorded at ~4 mM^8^, although significant seasonal variation is typical in ectotherms^6^. For comparison, in our experiments, we used 30 mM dextrose (D-glucose). In a *post mortem* system, these concentrations would likely have been higher than documented serum levels in reptiles, due to the haemolysis of red blood cells (see References 56-58 in the main manuscript).

**Synchrotron X-ray microprobe analysis.** Hard X-ray microprobe measurements were carried out at ALS bending magnet beamline 10.3.2 (2.1 – 17 keV) with the storage ring operating at 500 mA and 1.9 GeV. Samples were mounted on silicon nitride windows (Silson Ltd). Iron distribution and chemical speciation in *T. rex* tissues were investigated by micro-focused X-ray fluorescence (*µ*-XRF) mapping and near-edge X-ray absorption fine structure (*µ*-XANES) spectroscopy, respectively. Maps and spectra were recorded using a 7-element Ge solid state detector (Canberra). Elemental distribution maps (Fe, S, Ba) were collected at 10 keV on multiple tissue locations, using a 3 *μ*m $\times$ 3 *μ*m beam spot size, 3 *μ*m $\times$ 3 *μ*m pixel size with a 50 ms dwell time/pixel. Micro-XRF spectra were simultaneously recorded on each pixel in these maps. Maps were then deadtime corrected and decontaminated. Multiple spots were selected from each tissue region for further Fe K-edge *μ*-XANES analysis. Fe K-edge spectra were calibrated using a Fe foil with first derivative set at 7110.75 eV. Least-square linear combination fitting of the experimental spectra was performed using an extensive published beamline 10.3.2 spectral database of Fe standard compounds^9,10^ following procedures detailed elsewhere^11^. All data were processed using the LabVIEW custom software available at the beamline.

***Supplemental figures***


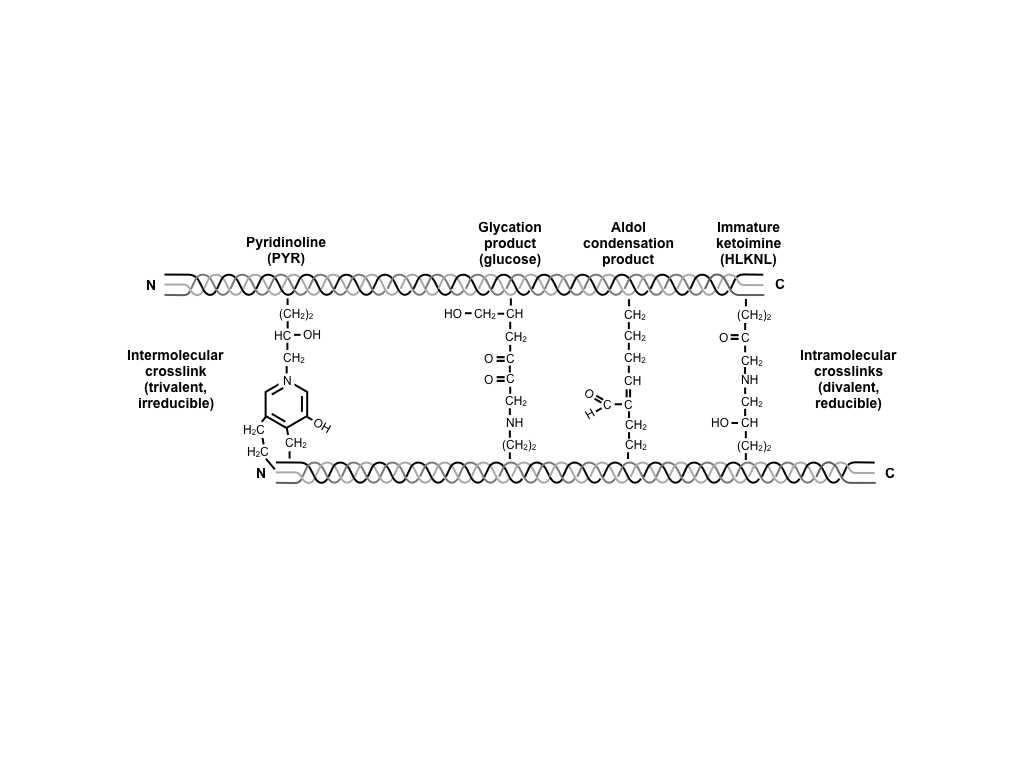


**Figure S1 | Intra- and intermolecular crosslinks in structural protein systems.** Divalent intramolecular crosslinks can form as the result of enzymatic or non-enzymatic pathways, such as transition metal-catalysed crosslinking. When reducing sugars are involved, such as glucose, advanced glycation end products can form. These common intramolecular crosslinks all contain carbonyls, which are directly testable using NaBH_4_ reduction methods^12^ in combination with Fourier-transform infrared spectroscopy. Divalent intramolecular crosslinks can mature into trivalent intermolecular crosslinks, which are irreducible. The latter class of crosslinks stabilises structural protein tissues, affording mechanical benefits such as stiffness, and preventing turnover, thus contributing to their accumulation with tissue age^13^.

**
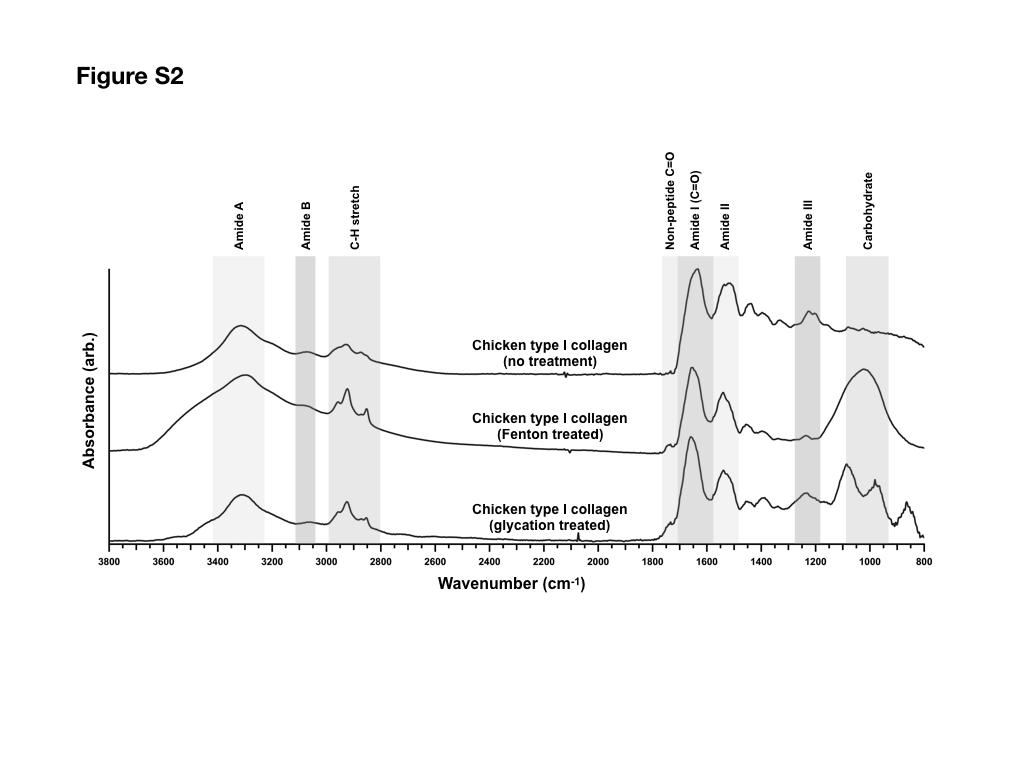
**

**Figure S2 | Full SR-FTIR spectra of untreated, Fenton treated, and glycation treated chicken type I collagen.** In all chicken samples, Amide I, Amide II, Amide III, Amide A, and Amide B bands are visible. Both crosslinking treatments introduce a small non-peptide carbonyl band (~1739 cm^-1^) and additional absorption in the region typically associated with carbohydrates (~1090-940 cm^-1^).
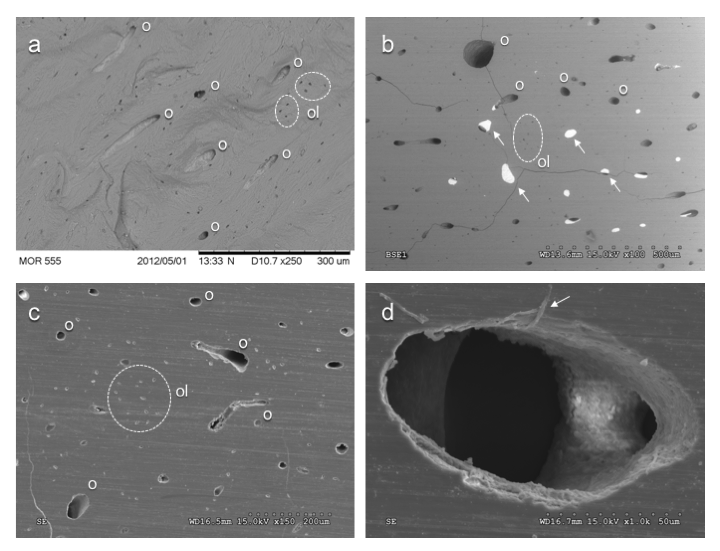


**Figure S3 | SEM images of USNM 555000 cortical bone. a**, Fracture surface showing clear features of osteons (o) predominantly in longitudinal section, osteocyte lacunae (ol; in dashed white circles), and fine texture consistent with mineralized collagen fibers in bone. Back-scattered (BSE) image. **b**, Polished (1200 grit) transverse section (BSE image) showing clear features of osteons and osteocyte lacunae. Mineral infilled osteons (white arrows) yield highly altered vessel structures (see Figures S4, S5), which were readily eliminated from SAXS, FTIR, and TEM analysis by careful preparation (sedimentation, washing, selection under microscope). Cracks are due to humidity/pressure changes and are an artefact of preparation. **c**, Polished (1200 grit) transverse section (secondary electron [SE] image) showing clear features of osteons and osteocyte lacunae. **d**, Highly magnified SE image of an osteon, showing fibrous texture at edges (white arrow), which was commonly observed in non-mineral infilled osteons in this specimen. This thin, fibrous coating inside the osteon structure is proposed to be the hollow, pliable vessel structures presented in Figure 2.

**
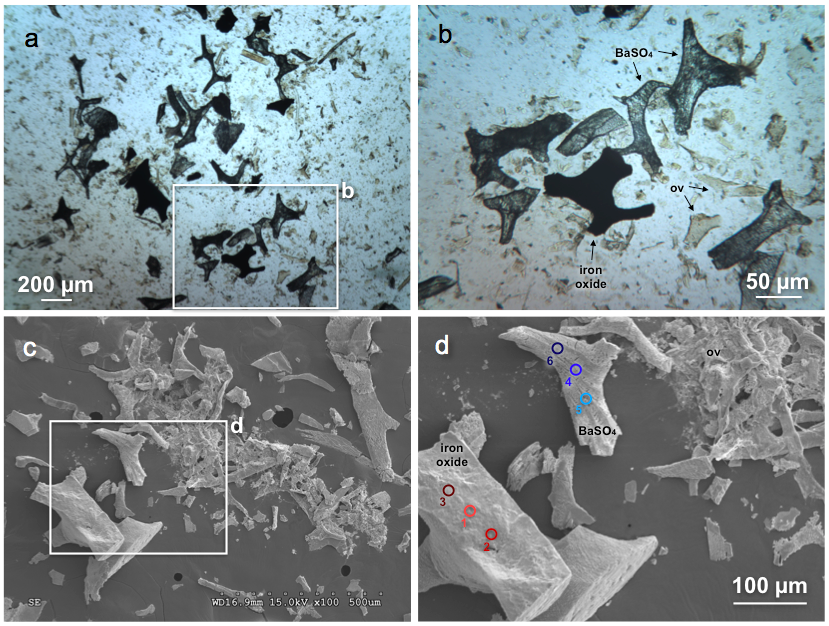
**

**
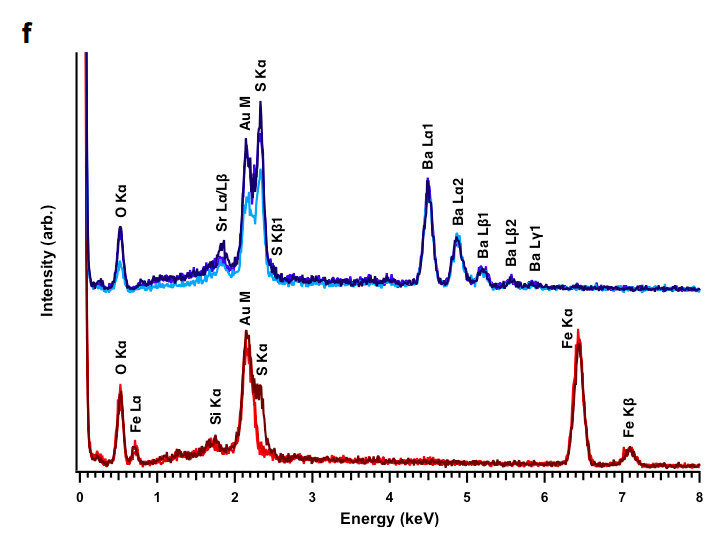
**

**e**

**Figure S4 | Images and chemical analysis of the organic, brown-hued *Tyrannosaurus rex* vessels and mineralised casts of the vessel system liberated upon tissue demineralisation. a**, Optical microscopy image of typical vessel fragments liberated from *T. rex*. **b**, Magnified image of white rectangle in **a**, depicting brown-hued, pliable, organic vessel (ov) fragments and two types of mineralised casts: opaque forms and semi-translucent forms. **c**, SEM image of similar vessel fragments. **d**, Magnified image of white rectangle in **c**, with three vessel fragment types identified and locations of EDS analysis in **e** explicitly identified. **e**, EDS analysis identified the opaque vessel casts as an iron oxide form (bottom, red) and the semi-translucent vessel casts as BaSO_4_ (top, blue).

**
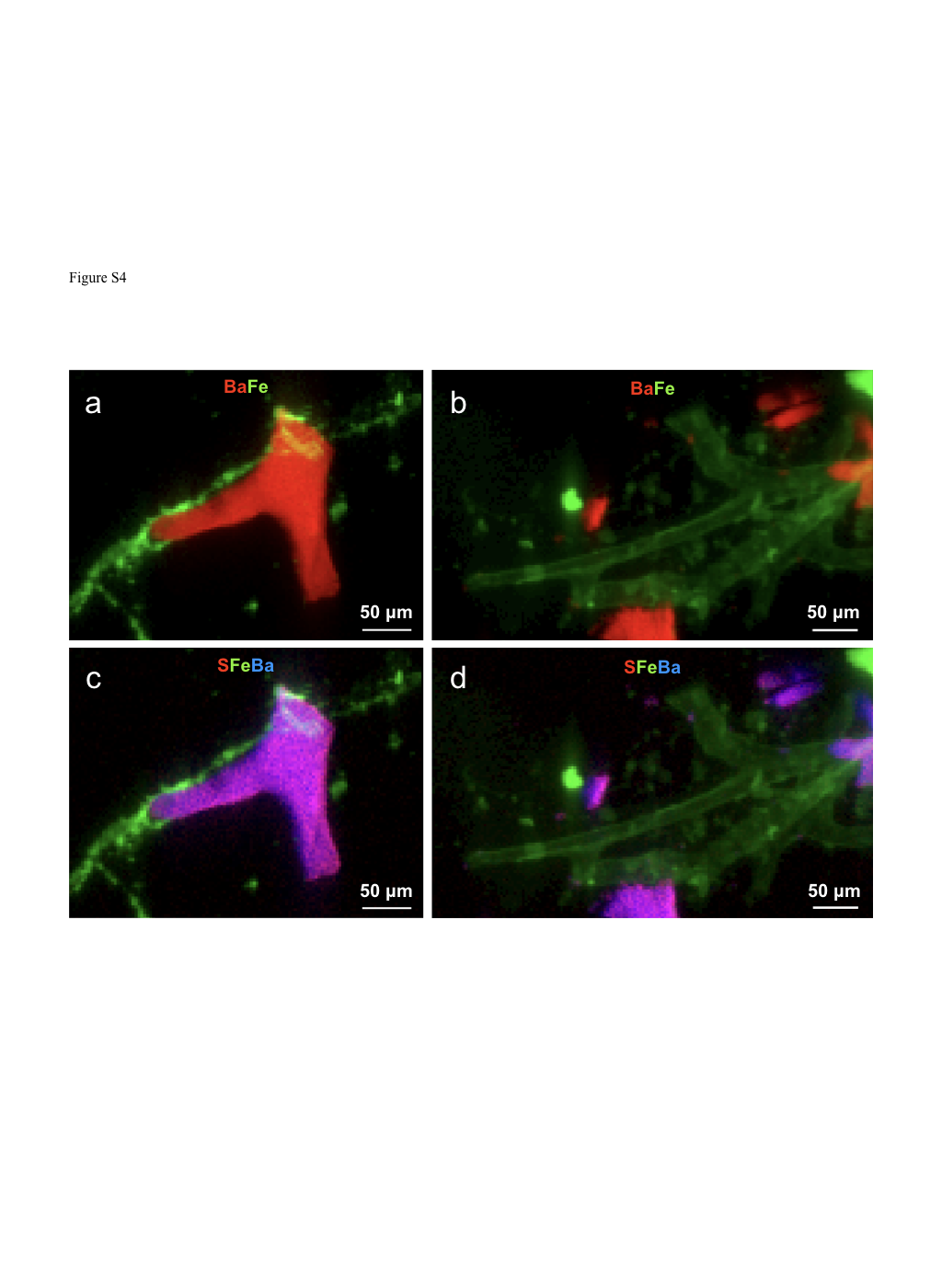
**

**Figure S5 | Micro-X-ray fluorescence (*μ*-XRF) mapping of organic and mineralised *Tyrannosaurus rex* vessel tissues reveals presence of multiple metals.** S, Fe, and Ba were the primary elements identified in the vessel samples. Composite maps of two (**a,b** show Ba and Fe) and three (**c,d** show S, Ba, and Fe) atomic species show that Fe is predominantly associated with the organic vessel tissues, whereas S and Ba are mainly associated with the semi-translucent mineralised vessel casts. Corresponding visible light microscopy images are provided in Figures 6 b,a, respectively.

**Figure S6 | SAXS analysis of the demineralised *T. rex* and extant chicken type I fibrillar collagen tissues to precisely determine d-spacing. a**, Radially integrated profile of *T. rex* vascular tissues. The first-order diffraction peak (red arrow) of fibrillar collagen was used to calculate a d-spacing value of 66.5 nm. **b**, Radially integrated profile of extant chicken type I collagen. The first-, third-, fifth-, and seventh-order diffraction peaks are identified (blue arrows). The first-order peak yielded a d-spacing of 65.0 nm. Insets: Whole pattern images with white arrows indicate first-order diffraction peaks (black bars are the beam blocker).


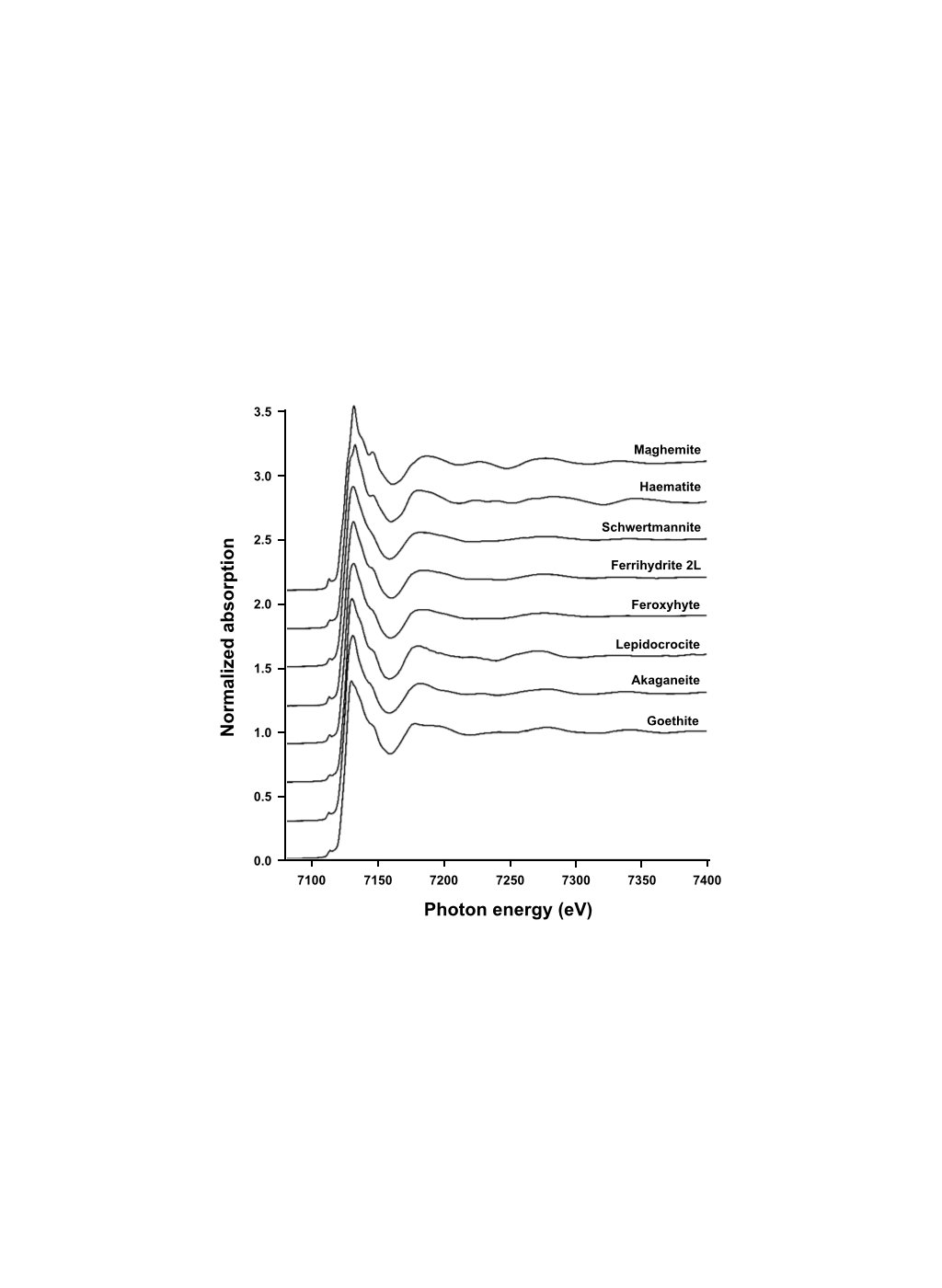


**Figure S7 | Fe K-edge XANES spectra of an example set of Fe^3+^ oxides in the ALS beamline 10.3.2 Fe spectral database.** Goethite (*α*-FeO(OH)), akaganeite (*β*-FeO(OH)), lepidocrocite (*γ*-FeO(OH)), feroxyhyte (*δ*-FeO(OH)), ferrihydrite (FeO(OH)$\boldsymbol{\cdot}$0.4H_2_O), shwertmannite, haematite (*α*-Fe_2_O3), and maghemite (*γ*-Fe_2_O3).

***References cited***

1. Buss, J.L., Neuzil, J., Gellert, N., Weber, C. & Ponka, P. Pyridoxal isonicotinoyl hydrazone analogs induce apoptosis in hematopoietic cells due to their iron-chelating properties. *Biochem. Pharmacol*. **65**, 161–172 (2003). (doi:10.1016/s0006-2952(02)01512-5)

2. Poňka, P., Borová, J., Neuwirt, J., Fuchs, O. & Nečas, E. A study of intracellular iron metabolism using pyridoxal isonicotinoyl hydrazone and other synthetic chelating agents. *Biochim. Biophys. Acta* **586**, 278–297 (1979). (doi:10.1016/0304-4165(79)90100-4)

3. Daddow, L.Y.M. A double lead stain method for enhancing contrast of ultrathin sections in electron microscopy: a modified multiple staining technique. *J. Microsc*. **129**, 147–153 (1983). (doi:10.1111/j.1365-2818.1983.tb04169.x)

4. Zimmermann, E.A., Gludovatz, B., Schaible, E., Busse, B. & Ritchie, R.O. Fracture resistance of human cortical bone across multiple length-scales at physiological strain rates. *Biomaterials* **35**, 5472–5481 (2014). (doi:10.1016/j.biomaterials.2014.03.066)

5. Ilavsky, J. Nika: software for two-dimensional data reduction. *J. Appl. Crystallogr*. **45**, 324–328 (2012). (doi:10.1107/s0021889812004037)

6. Dessauer, H.C. Blood Chemistry of Reptiles: Physiological and Evolutionary Aspects. In *Biology of the Reptilia*, vol. 3 (ed. Gans C. and Parsons, T.S.), 1-72 (Academic Press, 1970).

7. Hill, R.J., Konigsberg, W., Guidotti, G. & Craig, L.C. The Structure of Human Hemoglobin. *J. Biol. Chem.* **237**, 1549-1544 (1962).

8. Stacy, B.A. & Whitaker, N. HEMATOLOGY AND BLOOD BIOCHEMISTRY OF CAPTIVE MUGGER CROCODILES (*CROCODYLUS PALUSTRIS)*. *J. Zoo Wildlife Med.* **31**, 339-347 (2000). (doi:10.1638/1042-7260(2000)031[0339:HABBOC]2.0.CO;2)

9. Marcus, M.A. *et al*. Smelting of Fe-bearing glass during hypervelocity capture in aerogel. *Meteorit. Planet. Sci*. **43**, 87–96 (2008). (doi:10.1111/j.1945-5100.2008.tb00611.x)

10. Lam, P.J., Ohnemus, D.C. & Marcus, M.A. The speciation of marine particulate iron adjacent to active and passive continental margins. *Geochim. Cosmochim. Ac*. **80**, 108–124 (2012). (doi:10.1016/j.gca.2011.11.044)

11. Westphal, A.J., *et al.* MIXING FRACTION OF INNER SOLAR SYSTEM MATERIAL IN COMET 81P/WILD2. *Astrophys. J*. **694**, 18–28 (2009). (doi:10.1088/0004-637x/694/1/18)

12. Eyre, D. Collagen cross-linking amino acids. *Methods in Enzymology* **144**, 115–139 (1987). (doi:10.1016/0076-6879(87)44176-1)

13. Bailey, A.J., Paul, R.G. & Knott, L. Mechanisms of maturation and ageing of collagen. *Mech. Ageing Dev*. **106**, 1–56 (1998). (doi:10.1016/s0047-6374(98)00119-5)
